# Behavioral activation for children and adolescents: a systematic review of progress and promise

**DOI:** 10.1007/s00787-018-1126-z

**Published:** 2018-02-23

**Authors:** Faith Martin, Thomas Oliver

**Affiliations:** 1 Department of Psychology, University of Bath, Claverton Down, Bath, BA2 7AY UK; 2 University of the West of England, Frenchay Campus, Coldharbour Lane, Bristol, BS16 1QY UK

**Keywords:** Behavioral activation, Depression, Anxiety, Children, Adolescents, Systematic review

## Abstract

Behavioral activation (BA) effectively treats depression in adults, and shows promise in treating anxiety. Research into its application to children and adolescents is emerging. This review aimed to explore the scope of studies, current evidence of effectiveness and how the intervention has been delivered and adapted, to inform future research. A systematic review was undertaken searching PsycInfo, PubMed including Medline, EMBASE, and Scopus for terms relating to BA and children and adolescents. Two researchers scored abstracts for inclusion. Data extraction was completed by one researcher and checked by another. 19 studies were identified, across 21 published articles. 12 were case studies, with three pre–post pilot designs and four randomized-controlled trials. Case studies found early support for the feasibility and potential effectiveness of BA to address both anxiety and depression. The RCTs reported largely positive outcomes. Meta-analysis of depression scores indicated that BA may be effective; however, high heterogeneity was observed. Sample sizes to date have been small. BA has been delivered by trained therapists, doctoral trainee psychologists, social workers, or psychology graduates. Studies are uniquely in high-income settings. Adaptations include flexibility in content delivery, youth friendly materials, and parental involvement. There is some limited evidence to support BA as effective for young people. Feasibility and acceptability are supported. Fully powered trials are now required, with expansion to delivery in low- and middle-income settings, and detailed consideration of implementation issues that consider culture and environment.

**Electronic supplementary material:**

The online version of this article (10.1007/s00787-018-1126-z) contains supplementary material, which is available to authorized users.

## Introduction

Adolescent mental health is currently recognized as a global priority [1]. Mental health problems most commonly emerge in adolescence, and treatment at this life-stage can prevent problems from being maintained into adulthood, to avoid life-long mental distress and the individual, social, and economic costs that accompany this [1]. Depression in young people is a leading cause of disease burden [2], as is anxiety [3]. Indeed, the two frequently co-occur amongst young people [4]. Other mental health and developmental disorders are responsible for a large proportion of disability-adjusted life years lost amongst young people [2]. Effective, implementable interventions are then urgently required to address adolescent mental health. Behavioral activation has been found to be highly effective with adult depression [5, 6], yet its application with young people is in its infancy.

Cognitive-behavioral therapy (CBT) is recommended for treatment of children and adolescents [7, 8], along with interpersonal therapy, psychodynamic therapy, and family therapy [9]. However, access to treatment is low: in the UK, 55% of 12–15 years with mental health problems receive no service for these needs [10]. Across Europe, access is estimated at around 26% of youth receiving mental health services within 12 months [11]. In low- and middle-income countries, there are often no or very few available services [12]. Increased access to treatment is an important goal, and interventions that can be implemented at scale are required.

### Relevance of BA to children and young people

When considering appropriate interventions for child and young people to address their mental health difficulties, four issues must be considered:

1. The intervention is developmentally  appropriate: for instance, avoiding complex abstract thinking [13].
2. Clinically, the intervention can address both depression and anxiety disorders, owing to high co-morbidities [4], and potentially relevant to a range of other difficulties to maximise impact.
3. Culturally sensitivity of the intervention to values and conceptualisations of distress to be able to be implemented widely, in diverse settings and in multi-cultural contexts [14].
4. Scaleability of the intervention, with minimal resources to allow wide implementation, including non-specialist delivery [15].

Behavioural activation (BA) fits these requirements being feasible for adolescents owing to its simple, behavioural, concrete focus. It has an emerging evidence base for the effectiveness of trans-diagnostic use [5, 16]. It is individualised and culturally sensitive [17] and has been delivered by non-specialists [18]. It is an effective treatment for adult depression, being cheaper to deliver but not clinically inferior to traditional CBT [6]. As such, BA has significant promise for treatment of young people. Research into BA for children and adolescents is an emerging field.

### Overview of behavioural activation

BA originated as a behavioral treatment for depression, addressing the lack of positive reinforcement and excess of avoidance behaviors [19]. There are essentially two BA approaches in depression treatment: Washington BA [20] and brief BA treatment for depression or “BATD” [21]. The former focuses on the environmental context and how ineffective coping maintains depression [20]: depressed behaviors are seen as a way to cope, largely through avoidance, which is then directly addressed by engaging in helpful, alternative coping behaviors. It, therefore, requires a functional analysis of behaviors and tends to include a broader range of techniques than BATD. BATD seeks to increase reinforcement for non-depressive behaviors and decrease reinforcement for depression behaviors (such as avoidance) [21], however, does not require detailed functional analysis, and has emphasized the importance of the value placed by the individual of different behaviors.

Practically, the two types of BA share many features: both are based on the fundamental premise that behavioral avoidance is central to depression and both aim to increase adaptive activities that reduce depression, decrease activities that maintain depression, and problem solve barriers to reward [22]. Both forms of BA have core techniques of self-monitoring and activity scheduling [5]. Eight techniques have been identified as components of BA: activity monitoring, activity scheduling, contingency management, values, and goal assessment, skill training such as problem solving, relaxation, targeting verbal behaviors, and targeting avoidance [19].

### Defining BA when applied beyond depression

BA was specifically designed to address depression. The extent to which behavioral therapy for non-depression problems can be called BA is unclear, rather than standard behavioral therapy. BA has increasingly been discussed in relation with anxiety disorders [23, 24]. Depression and anxiety share similarities, with negative reinforcement and avoidance in common [25]; therefore, BA may be applied in anxiety disorders. Classic behavioral therapy for anxiety often uses exposure-based classical conditioning theory to extinguish anxiety responses, whereas BA rests on operant conditioning principles and a functional contextualized perspective to address reinforcement [5]; therefore, the underpinning logic can define what is and is not BA in relation with anxiety.

For interventions, seeking to address problematic behaviors, in autism, for example, the picture may be less clear, as some behavioral therapy interventions use operant conditioning principles to shape behaviors, reinforcing non-problematic behaviors [for example, 26]. This differs from BA, as the behavioral shaping is completed for its own end, rather than to change behavior as a mechanism to influence mood state and psychological outcome [19]. For the purposes of this review, behavioral therapies using exposure only, or designed with the specific goal of altering undesired behaviors are not conceptualized as BA.

### Objectives of review

A recently published systematic review with meta-analysis by Tindall and colleagues [27] focused on the effectiveness of BA to treat adolescent depression. The review reports that BA may be effective in the treatment of depression in young people; however, caution is required given a small number of studies and their methodological limitations. The review focuses solely on the treatment of depression, for which BA was originally designed. However, BA has been applied to other conditions with adults and the presence of such developments for treating young people is not yet reviewed.

It is important to summarise the progress of BA as applied to young people, to help direct future research globally. Summarizing and analysing the details of what has been delivered, to whom, where, and how may guide researchers in the ongoing development of the intervention. A detailed review is required not only in relation with the potential effectiveness of the intervention, but also for how the intervention can be delivered and implemented, which should be considered early in the intervention development process [28, 29]. As such, this review seeks to extend beyond the scope Tindall and colleagues paper [27], with wider objectives.

The first objective of this review is to analyses the state of the science in relation with empirical basis and evidence base of BA as a treatment for children and young people, specifically to review studies reporting any quantitative data from children or adolescents who have undergone behavioral activation to provide initial analysis of outcomes on psychological wellbeing (including depression and anxiety), regardless of comparison group or study design, and the current reach and scope of the research. This includes investigating.

1. The study designs used.
2. The types of difficulties BA has been used for with children and young people.
3. The reach of the research to date in terms of participant characteristics, particularly in terms of cultural setting and ethnicity.
4. Evidence to date of effectiveness and/or adverse effects on depression and anxiety outcomes in children or adolescents.

The second objective is to explore how BA has been delivered to children and young people, including the location of delivery, therapist type and training, mode of delivery and number of sessions.

The third objective is to identify what has been learnt about adapting BA for children and young people, particularly in relation with parental involvement, and offer recommendations for future research. These objectives are essential to analyze knowledge to support future development of the intervention and its implementation.

## Methods

There is no published review protocol; details of the methodology are provided here.

### Eligibility criteria

Relating to study aims and as research into BA with children and young people is in its early stages, this review sought to identify all papers that had explicitly delivered a BA focused intervention, regardless of the study design, presence of comparison groups, length of follow-up, or the details of the psychological difficulty. Inclusion limits were: published papers with participants must include those up to the age of 19 years, based on the World Health Organization definition of “adolescence” [30], and interventions must use BA but not as part of a broader cognitive-behavioral or other therapeutic approach. Exclusion criteria were interventions which used behavioral therapy to reduce problematic behaviors only, prevention interventions, and purely qualitative papers.

### Information sources

PsycInfo, PubMed including Medline, EMBASE, and Scopus were searched. No limits relating to date or language were included. Search was complete up to the end of the 1st week of December 2017. References included in studies identified as relevant were also searched for potentially relevant papers.

### Search

Search terms were “Behavioral activation”, “behavior therapy” or “behavioral therapy” (also capturing UK spelling), AND child, children, adolescent, adolescence, young person, young people, youth, teen, or teenager, with search terms in title or abstract only. This highly sensitive search was necessary to ensure all behavioral therapy interventions, that may include behavioral activation interventions, were captured.

### Study selection

Duplicate records were removed. Abstracts of all identified studies were screened for eligibility by two researchers. Results were compared and final inclusions at abstract stage made. Differences in decisions to include/exclude were noted and discussed until agreement reached. Full texts for all included abstracts were sought. Full texts were inspected by one researcher, who proposed exclusion of the studies, where no relevant from the results could be found or where the intervention’s full description revealed it to be not BA. The second researcher checked these decisions.

### Data collection process

Full texts were inspected by one researcher, with data extracted into a prepared form. A second researcher checked one-third of the data extraction reports for accuracy and completeness (picked at random from the database). Errors or omissions were noted, and if differences were noted in two or more checked papers, it was planned to check data extraction for all included studies. As few additions or edits were made, no further checking was undertaken.

### Data items

Data were captured covering details of the sample (including sample size, participant ages, where drawn from, psychological difficulties assessed), intervention description (covering general description and inclusion of listed identified BA technical content, for example, functional analysis, with an option for “other” content not included in our list), study design, results relating to psychological outcomes, and any information relating to the adaptation of BA for use with children and young people.

### Risk of bias in individual studies

The Cochrane Collaboration’s tool for assessing risk of bias (CCAR) at an individual study level was used for the RCTs only [31, 32].

### Summary measures

Meta-analysis was conducted with data reported in randomized-controlled trials (RCT). Where multiple scales for the same outcome (e.g., a number of depression measures) were reported, measures shared between studies were selected where possible. The main summary measure is standardized mean difference, with 95% confidence intervals (CI).

### Synthesis of results

A random effects model was used to synthesize results, owing to high expected (and observed) heterogeneity. Heterogeneity was assessed with the calculation of *I*^2^ (value of 75% or more seen as “high” [31]).

### Risk of bias across studies

A summary of risk of bias across studies was made based on the CCAR, as above.

## Results

**Fig. 1:**
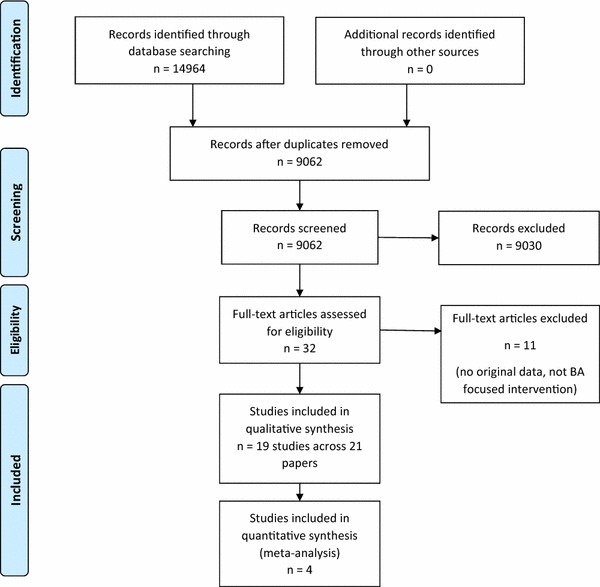
Screening and inclusion process for identified studies

The flow of studies through the review is illustrated in Fig. 1, in accordance with reporting guidelines for systematic reviews [33].

### Study selection

Agreement was achieved between the two researchers examining abstracts for inclusion/exclusion on 8360/8531 (98%) abstracts and 100% agreement was achieved at full-text stage. 19 relevant studies, published across 21 papers, were included in the review.

Ten articles were excluded at full-text stage, as no original data were provided [34–37], all participants were over 18 years [38], or the intervention was not BA, being either broader cognitive-behavioral therapy or narrowing behavioral shaping [39–44].

Of note, two interventions describing themselves as BA were excluded. Both were with children with ADHD and sought to modify undesirable behaviors [41, 42]. It is difficult to distinguish whether these studies are truly BA, or are simply behavioral therapy applied to ADHD, as the hallmark features of BA relate primarily to depression. As defined in this review, however, these studies are not BA, as they seek only to modify undesirable behaviors for that end, rather than to change behavior to then affect psychological outcomes.

### Study designs of included studies

The 19 relevant studies included 12 case studies/case series [45–56], three uncontrolled pre–post designs [57–59], and four RCTs [60–63].

Data from the one RCT [62] were also published in a paper focusing on mediation analyses [64], and in a paper reporting neuroimaging data [65]. All three papers were consulted for novel details for data extraction. Weersing and colleagues reported their RCT in two publications [37, 63]: data from 2017 were extracted only as these covered details from the 2016 publication. The data reported across from individuals in the case studies by Pass and colleagues [54–56] are also included in the pre/post study [59].

Table 1 provides details of study designs, participants, setting, intervention, comparator, outcome measures, and data collection points. More details in given across the online resources: Online Resource 1 details the study designs, types of difficulties addressed, and further details of participant characteristics, which are summarized here.

**Table 1:** Descriptive summary of study characteristics

Study references	Design	Subjects	Intervention	Comparator	Outcome measures	Setting	Data collection points
[45]	Case study	One 17 year old female with depression	10 individual sessions with graduate student CBT trained	None	BDI	USA. Clinic	Pre, session 6/8 BATD, last session
[46]	Case studies	4 young people, 2 European American, 1 African–American, 1 mixed ethnicity (European–Korean) (2 female) with depression based on Diagnostic Interview for Children and BDI-II score. Age 12–18	12 individual sessions with Clinical Psychology doctorate student	None	DISC and BDI-II	USA. Clinic	Baseline, immediately post-treatment and follow-up (varied by participant from 1 to 3 months). BDI-II also at every session
[47]	Case studies	2 (1 female) with depression or anxiety based on semi-structured interview. Age 7–17	8 individual sessions. Delivered by on-site Mental health clinical staff	None	SCARED, CDI	USA. Pediatric clinics	Pre treatment, end treatment (8 weeks), 12 week post-treatment, 24 week follow-up
[48]	Case series	5 (4 completed, 3 female) young people. 2 White, 1 African–American, one African immigrant, one Asian–American. Met criteria for depression or anxiety based on ADIS-IV-C. Age 12–14	13 group sessions at school with Clinical Psychologist and trainee clinical psychologist	None	ADIS-IV-C, MASC-C/P, CES-D-C/P	USA. Large state middle school	Pre and post
[49]	Case study	Case descriptive composite but scores from one 17 year old male. Diagnosed with Major depressive disorder	14 individual sessions with therapist	None	CDRS-R and SMFQ	USA. Clinic	Pre and then repeated, but score details for after baseline not given
[50]	Case series	5 females, scoring in depression range on CES-D. Age 13–18	10 sessions with individual with social workers	None	BDI	Australia. Adolescents in rural area	Baseline, 2 weeks, 3 weeks, 6 weeks and end of treatment (10 weeks)
[51]	Case series	3 (1 female) African America 13–17 year olds with major depressive disorder on K-SADS	Invited to 14–17 individual sessions with clinical staff trained in BA	None	K-SADS, CDRS-R, BDI-II, CGI-S	USA. Urban hospital	Baseline, end of treatment. CDRS-R at 9 weeks also and weekly BDI-II
[52]	Case series	11 (2 female) with depression on the CDRS-R. Age 8–12	9 sessions -3 sessions each for child, carer, and both together. Delivered by doctoral clinical psychology student	None	CDRS-RCDI	USA. School setting	Pre-, post-control treatment, post BA, 2 month follow-up
[53]	Case series	5 (1 female) 12–13 year olds with anxiety and depression relating to bullying, diagnosed using ADIS-IV-C, SCARED, CES-D	14 individual sessions with graduate psychology students	None	ADIS-IV-C to create CSR. SCARED, CES-D	USA. School setting	Baseline and post-treatment (interval not given)
[54]	Case study	Female age 15 referred with depression symptoms	9 individual sessions with clinical psychologist (1 review)	None	RCADS, RCADS-P (parent version)	UK. Mental health clinic	Session by session and 1 month follow-up
[55]	Case study	Female age 16 referred with depression symptoms	9 individual sessions with clinical psychologist (1 review)	None	RCADS, RCADS-P (parent version)	UK. Mental health clinic	Session by session and follow-up at 6 weeks
[56]	Case study	Female age 16 with depression symptoms	8 individual sessions with psychology assistant without specialist clinical training	None	RCADS, RCADS-P (parent version)	UK. Mental health clinic	Session by session and 1 month follow-up
[57]	Pre/post	6 young people (3 female) with major depression (K-SADS, CDRS-R). Age 14–17	Maximum 22 individual sessions. Delivered by 2 doctoral level faculty members and one senior-level graduate student	None	K-SADS, CDRS-R, BDI-II	USA. Clinic setting	First session and post-treatment. BDI also weekly
[58]	Pre–post	28 (19 female) 14–17 year olds with diagnosis major depressive disorder (K-SADS, CDRS-R). Ethnically diverse sample	Maximum 22 individual sessions. Delivered by 3 doctoral level psychologists, two advanced graduate students	None	CDRS-R, BDI-II, CBCL and CGI-S	USA. Clinic setting	Baseline, mid-point, end of treatment, 3-month follow-up, 6 month follow-up
[59]	Pre–post	20 (18 female) 14–17 year olds referred to outpatient clinic for depression treatment, 10 with co-morbid anxiety diagnosis	6–8 1 hour weekly one-to-one sessions, with a further review sessions. Parents invited to 3 sessions. Delivered by Psychology assistants and Clinical Psychologist. Trained in BATD by Lejeuz	None	RCADS, RCADS-P (parent version)	UK. Mental health clinic	Session by session and 1 month follow-up
[60]	RCT	35 (25 female) 12–14 year olds with current clinical or sub-clinical difficulties with depression or anxiety. Ethnically diverse sample (13 Hispanic, 15 African–American, 5 White non-Hispanic, 2 multiple) with broad range of family incomes	*n* = 21. 10 group sessions, two individual sessions. Each group delivered by two therapists from one clinical psychologist, four psychology graduate students, two school counsellors	Wait-list control, *n* = 14	ADIS-IV, CDRS-R, CGI-S, SCARED, CES-D	USA. School setting	Pre, post, 4 month follow-up
[61]	RCT	60 (38 female) 12–18 year olds with depression diagnosis based on CDRS-R	*n* = 35. 14 individual sessions with either psychology post-doctoral fellows or social worker	Evidence-based intervention, *n* = 25	CDRS-R, CGI-S, SMFQ	USA. Metropolitan area. Recruited via primary care and mental health	Pre-, end of treatment, 6 month follow-up, 12 month follow-up
[62] and [64] and [65]	RCT	118 (45 female) first year university students aged 18–19 with sub-clinical depression —BDI-II score at least 10 and CIDI interview confirm sub-clinical level	*n* = 62. 5 individual sessions with doctoral research fellow with CBT experience	Control—inactive, no therapy or contact, *n* = 56	BDI-II, Japanese version	Japan. University (first year students)	Pre–post
[63]	RCT	185 (107 female) youths aged 8–16 meeting criteria for at least one of depression, separation anxiety, generalized anxiety disorder, social phobia, major/minor depression or dysthymic disorder using CGI-S	*n* = 95. 8–12 individual sessions with master’s level therapist	Assisted referral to care, *n* = 90	CGI	USA. Pediatric clinic	Baseline, post intervention at week 16

*BDI* beck depression inventory [66], *BDI-II* beck depression inventory-II [67], with the Japanese version [68], *DISC* diagnostic interview schedule for children [69], *ADIS-IV-C* anxiety disorders interview schedule for DSM-IV—Child Interview, used to create CSR—Clinical Severity Rating [70], *MASC-C/P* multidimensional anxiety scale for children—child and parent scale [71], *CES-D-C/P* centre for epidemiologic studies depression scale for children—child/parent reports [72], *CDRS-R* children’s depression rating scale—revised [73], *SMFQ* short moods and feelings questionnaire [74], *K-SADS* kiddie schedule for affective disorders and schizophrenia [75], *CGI-I/S* clinical global impressions scale—impairment/severity [76], *SCARED* screen for childhood anxiety related emotional disorders [77], *RCADS* revised child anxiety and depression scale, including RCADS-P as parent version [78], *CBCL* child behavior checklist for ages 6–18 [79]

### Types of difficulties BA used to address

BA has been used to treat depression, anxiety, and co-morbid anxiety and depression. Major depressive disorder, clinical, and sub-clinical depression have been addressed. Young people with panic, social anxiety/social phobia, generalized anxiety disorder, and/or separation anxiety have been included in the studies, with some inclusion of participants with co-morbidities amongst these difficulties.

### Participant characteristics

Studies have solely been conducted in high-income settings: 13 in the USA, 4 in the UK, 1 in Japan (published across three papers), and 1 in Australia. Several of USA-based studies described ethnically diverse populations and engaging with low-income, African–American families. BA has been offered to a total of 313 adolescents [182 (58%) female].

### Risk of bias

Table 2 provides a summary of risk of bias analysis for the individual RCT studies. For just one study, an online registered protocol was available (https://clinicaltrials.gov/ct2/show/NCT01147614), confirming no selective reporting.

**Table 2:** Summary of risk of bias analysis for individual studies

Domain	Chu et al. [60]	McCauley et al. [61]	Takagaki et al. [62]	Weersing et al. [63]
Random sequence generation	+	+	+	+
Allocation concealment	?	+	+	+
Blinding participants and personnel	–	–	–	–
Blinding outcome assessment	+	+	+	+
Incomplete outcome data	+	+	+	+
Selective reporting	?	?	?	+
Other sources of bias	+	+	+	+

Key: “+” low risk of bias, “- “high risk of bias, “?” unknown/unreported

Overall, risk of bias was high in relation with the challenges of blinding participants to their allocation to treatment or control group. Attrition bias between treatment and control groups was not analysed (presumably due to generally small sample sizes); therefore, it is not clear if participants’ were more likely to be retained in either condition, nor if there were particular participant characteristics linked to participant attrition or incomplete data. Studies did generally use blind assessors for outcome and random sequence generation for allocation was performed in all studies.

### Summary of outcomes of individual studies

Online Resource 2 provides detail of reported outcomes from all studies, together with outcome measures used, data collection points, and comparator group details, where applicable.

A range of measures have been used, with BDI and CDRS-R measures most commonly used.

Control groups received no control intervention [62]; wait-list control [60]; or referral to mental health care [37]; with just one study using an active control comparison of evidence-based intervention, being either cognitive-behavioral therapy or interpersonal therapy [61].

Although results are generally reporting data in favor of BA potentially having an effect on reducing symptoms across the disorders, the vast majority of studies are case studies and uncontrolled. 15 of the studies reported some positive results (statistically significant change in severity scores or movement for majority of cases from scores indicating clinical caseness to non-caseness).

### Synthesis of results

Examining only the four RCTs [37, 60–62], effect sizes (Hedges g) were calculated for each measure at post-test (not papers reported data for later follow-up periods) comparing control and BA group scores, detailed in Online Resource 3. Sample sizes are all small, with the largest study randomizing 185 people [37].

**Fig. 2:**
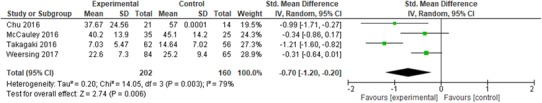
Random effects meta-analysis of depression scores across included RCTs (*n* = 4)

Meta-analysis was conducted with data from the RCTs. Only one RCT reported anxiety measures, as such meta-analysis focused on depression scores. Three of the four studies used the same measure (CDRS-R); therefore, data for this was measure used where possible, with the BDI scores used for the remaining study. It must be noted that these studies used different comparators: two offering no active intervention (wait-list or no intervention), one referral to mental health services, and one active treatment. The effect of BA was large, with a pooled standardized mean difference of − 0.70 (95% CI − 1.20, − 0.20) [80], see Fig. 2. This demonstrates a statistically significant difference in favor of BA; however, heterogeneity was high: *I*^*2*^ = 0.79.

### BA intervention content

Online Resource 4 details the intervention content, based on what is explicitly referred to in the studies. These categories are derived from literature reviews [19] summarizing what BA consists of (first eight columns) and common themes from the intervention descriptions. Intervention content differs in complexity, with interventions included three categories of content [52], all the way up to 10 [61]. Activity monitoring and scheduling were almost always present. Functional analysis was observed in descriptions of the interventions in 14 of the studies. Functional analysis is a wide category, including both simple analysis of antecedents and consequences of behavior in a basic three item cycle, as seen several studies using BATD, for example [45, 59]), and more complex, personalized analysis seen in BA derived from the “Washington BA” approaches [20] as seen in studies by Chu and colleagues, for example [48], using more detailed analysis of avoidance patterns and alternative responses.

### Details of delivery approaches for BA interventions

Online Resource 5 summarizes location, therapist, mode, and number of sessions offered in each study. Four studies were delivered in a school setting, whilst all others were at an outpatient clinic. Therapy was delivered by clinical psychologists, doctoral level psychologists, trained therapists, or social workers. Only one study used non-specialist trained staff to deliver the intervention: a psychology assistant [56]. No studies used trained lay people. All studies used face-to-face delivery of interventions, with a mixture of group and one-to-one settings (three group, one mixed group and individual, remaining 15 individual). Between five and 22 sessions were offered, and most (12 studies) descriptions included the offer of joint sessions with parents.

### Adaptions of BA for children and young people

Parental involvement was the most common adaptation of BA for young people. This included parents attending sessions, workbooks for parents, and involvement of parents in supporting the young person to complete their chosen activities. Family engagement was to offer support, address barriers and create positively reinforcing interactions [47, 49, 51, 54]. This addresses also the challenges that young people have relating to how much they control their environment and how their time is spent. The wider impact that changing behavior in a young person may have on their family and other social systems needs to be considered [57].

Some studies suggest intervention with fewer techniques which are simpler to deliver and follow [37, 47]. Other studies used a greater range of techniques including skills and practice sessions to improve communication between parents and young people [45]. Problem solving skills may be particularly developmentally appropriate for young people [49, 54]. It can be presented with acronyms to simplify and aid remembering, e.g., use of “ACTION” to present problem solving [57]. Active practice of these skills in sessions, including role play, supports learning [49]. Concrete goal setting is encouraged in one study, with awareness that must be realistic in choice of goals that young person can access resources required, for example, transport if the goal is to go to the gym [57]. It has been found useful to generate a list of developmentally appropriate activities for young people to engage in and how this could be further reinforced, including joint parent–child activities for younger children [45]. Alternatively, using developmentally appropriate prompts of life areas relevant to young people was found to help structure goal setting, e.g., “Me”, “Things that matter”, and “The bigger picture” [54].

Rather than structured psychoeducation, a more flexible approach is useful, for example, tailoring worksheets and handouts to the individual in a collaborative, client centered way, to maintain engagement [49, 57]. Handouts or workbooks were developed to be attractive, engaging, and developmentally appropriate and tailoring of language used to an appropriate register [47, 50, 54, 56, 57].

Homework non-completion may require attention, focusing on exploring benefits of therapy and being flexible about homework completion where needed to maintain engagement [56]. Some interventions were delivered at school, to allow easy attendance for the young person and use school administration systems to support intervention delivery [48].

An important adaptation is development of interventions to address both anxiety and depression, through common mechanism of reducing avoidance, as comorbidity is high [37, 47, 48, 53, 60]. One intervention particularly focused on the impact of the common experience of bullying on young people, tailored to ensure confidentiality was well understood by all to create an environment where they felt safe to engage in the intervention [53].

## Discussion

This systematic review explored the study design, application, reach, and evidence of effectiveness for BA in children and adolescents, in addition to examining how BA has been delivered and what adaptations have been made to BA to increase suitability for application to young people.

Comparing this review with the recent systematic review and meta-analysis focusing on the effectiveness of BA for treating depression in young people [27], there are clear differences in methods that underpin the differences in reported results and discussion. The main differences in methodology are that this review did not focus specifically on depression, did not search the grey literature, did not search CINAHL (owing to lack of access to this database), but did include case studies and data extraction extended to more detailed descriptions of intervention delivery, content, and adaptations. This reflects the differences in the two reviews’ objectives. This review then included studies not only focusing on depression but also on anxiety and did not include two studies seen in Tindall’s review that are not published in peer-reviewed journals [81, 82].

Research into BA with young people is at an early stage, reflected in study numbers, design, and quality. The reach of the research to date is limited. Although depression and a range of anxiety disorders have been included, the sample size and diversity of young people involved in the research remain very limited. Included studies do not cover children and adolescents with medical problems, where rates depression and anxiety are higher than in a physically healthy population [83]. Despite growing acknowledgement of the importance of young people’s mental health globally [1], studies have all taken place in high-income countries. Whilst this is not surprising in terms of access to funding for early stage research, it is now important to explore how this intervention could be applied in other contexts. It is not clear to what extent BA is feasible in situations of poverty and impoverished environments, where details of and access to positively reinforcing activities may be very different, and in cultures were the identity, social development, and role of children and young people may differ.

The majority of studies are case studies, which have more recently informed RCTs. As such, it is not possible to draw firm conclusions regarding the effectiveness of BA to treat child and adolescent mental health problems. However, the data reported are typically promising as to the intervention’s potential effect and meta-analysis for depression indicated effect in favor of BA, although with some methodological limitations. The feasibility of this approach with this age group is supported.

The review found that BA has been delivered by at least graduate level psychologists if not fully qualified therapists or health and social care workers. BA has been delivered to adults by lay staff, increasing its potential scalability [84]. Meeting the demand for effective therapies for children and adolescents is a priority, yet nothing is known currently about the use of lay therapist in BA’s delivery to this group. Given that many of the adaptations made to BA revealed in this review are to make it flexible in delivery to engage young people, it is not clear whether lay delivery would be possible, or if a higher level of technical skill will be required to tailor treatments to children and adolescents.

BA has been primarily recruited to and delivered through mental health and education systems, with just three studies recruiting via general community routes [46, 57, 58]. Many young people with mental health problems may be disengaged from education and not joined up with mental health services [10, 12, 85]. Implementing BA outside of existing systems may present greater challenges to its feasibility, in terms of recruitment and retention but also in relation with BA mechanisms. Based on increased positive reinforcement from activities, BA requires that the participant can access and engage in such activities. Availability of social activities, for example, may be scant for marginalized or impoverished young people. To address implementation challenges, researchers may need to explore and intervene with what activities are actually available in the wider environment and perhaps include in the overall intervention plan development or connections to community youth groups, for example.

What has been delivered as “BA” and how it was delivered are varied. Some descriptions of intervention content are poor in detail and quality. Despite several studies reportedly using the same intervention, descriptions of the interventions vary—for example across the studies conducted by Chu et al., only one makes specific reference to including relapse prevention [60]. This difficulty in reporting is well acknowledged [86], and researchers in behavior change interventions have developed taxonomies to attempt to improve descriptions [87]. A similar approach for mental health interventions may be of use, to encourage authors to clearly and fully describe the essential techniques used in their interventions and to allow readers and systematic reviewers to better classify intervention content.

The optimum content of BA for this population remains unclear. “Functional analysis” content is seen in many interventions reviewed here, but varies significantly in its emphasis, depth, and complexity. The inclusion of functional analysis, explicit addressing of avoidance, and targeting of verbal behaviors, for example, may or may not improve outcomes. This issue is also seen in the BA for adults’ literature; for example, whilst avoidance is explicitly targeted in several BA interventions, there is little direct support for this over and above activity scheduling [19]. A review, with sub-group analysis, of the adult BA literature concluded that there appeared to be no impact on outcome as to whether in-depth functional analysis was included or not [5]. As research develops, optimizing the intervention to be efficient and cost-effective will be necessary.

Adaptations made to BA to use with young people have revealed themes in ensuring flexibility of delivery, partly to maintain engagement, and designing tailored materials and lists of activities that are developmentally appropriate. BA is found to be feasible with children and adolescents in terms of the cognitive demands and developmental appropriacy: participants could follow materials, generate lists of goals, and engage in the techniques offered.

Parental involvement is a key issue in interventions with children and adolescents. Overall, inconsistent support has been found for parental involvement in relation with cognitive-behavioral therapy outcomes [88]. This review highlights its importance to allow the young person to meaningfully engage in therapy through being able to carry out scheduled activities with parental support and resources, where needed. The necessity of this will vary by individual’s context and research could develop brief, clear protocols to assess if/what parental involvement may be required for each young person. Such protocols may be essential for delivery of BA by non-specialists. Given the direct interaction with the environment that BA demands, there may be a ceiling of change where parents, and wider social environments are not taken into account: BA may be able to impact positively, but reach a ceiling where the young person cannot do planned activities, or continues to experience conflict in the home that relates to presenting the depression or anxiety. Indeed, barriers and facilitators to implementation of BA for children and adolescents have received little attention in the literature to date.

### Limitations

This review included only published studies, studies with at least basic reporting of outcomes data and studies focusing on treating, rather than preventing, mental health difficulties. We did not contact authors to obtain further unpublished information. The extent of publication bias towards positive results is unknown.

Three excluded studies described BA style interventions, but did not provide any participant outcome data. Spirito et al. used BA techniques with children with cystic fibrosis to address anxiety [36]. Davidson et al. explored the use of web-based BA with adolescents with depression [34]. Welsh et al. discussed the potential application of BA for adolescents at risk of psychosis [35]. These areas of clinical health, online delivery, and psychosis represent further potential areas of development for BA research. This review does not include any qualitative findings from work with these client groups.

The methodological limitations of the studies reflect the early stage of research into BA for this population. Case studies provide little information regarding effectiveness; however, these reports together with the pre/post and RCT reports offer detailed information regarding the design of the intervention itself.

Meta-analysis was completed; however, results must be interpreted with caution owing to high heterogeneity, risk of bias, differences in control conditions, and one study used a different depression outcome. Risk of bias was particularly relevant in relation with the general lack of published protocols, meaning that it is not possible to evaluate reporting bias. Furthermore, owing to the nature of the research, concealment of allocation from participants and those delivering the intervention was problematic in the RCTs, particularly those that did not use an active intervention in the control group. Meta-analysis was not possible for the application of BA to anxiety for young people, as only one RCT included this outcome.

## Conclusions

This review provides a detailed examination of the state of the science and details of intervention content, to inform future work into intervention design and methodology of future studies. Whilst there is support for the feasibility of the intervention, and substantial recommendations for how this can be delivered, there is insufficient evidence to draw conclusions regarding effectiveness, although initial data support effectiveness for depression. Recommendations for future research include the need to develop and test BA for young people in low- and middle-income countries and with a range of cultures, paying attention to how BA may need to be further adapted for young people where their social development and role may differ to those in high-income countries. Methodologically, it is now necessary to move to fully powered RCTs to explore effectiveness in detail. Alongside this, issues of how to optimize intervention content to ensure maximum efficiency and cost-effectiveness must be considered, to aid scalability. To extend the potential practical promise and implementation of BA, it is important to explore issues such as BA can be effectively delivered to children and adolescents using lay therapists and to document and address barriers and facilitators to implementation, alongside effectiveness research.

## Electronic supplementary material

Below is the link to the electronic supplementary material.

Supplementary material 1 (DOCX 112 kb)
