# Supplementary material for: Behavioral activation for children and adolescents: a systematic review of progress and promise
Source: Eur Child Adolesc Psychiatry. 2018 Feb 23;28(4):427–41. doi: 10.1007/s00787-018-1126-z (PMC6445819; doi:10.1007/s00787-018-1126-z)
Supplement: Supplementary file 1 — Supplementary material 1 (DOCX 112 kb) [file 787_2018_1126_MOESM1_ESM.docx]

**Online Resource 1 Study design, target difficulties and participant characteristics**

| **Study reference** | **Design** | **Target difficulties** | **Inclusion criteria** | **Recruitment source and numbers** | **Setting / other participant characteristics** | **Sample size and gender** | **Number offered BA** |
| --- | --- | --- | --- | --- | --- | --- | --- |
| (Ruggiero, Morris, Hopko, & Lejuez, 2007) | Case study | Depression | None. Adolescent with history of child abuse. | Selected clinical case | USA. No details re: clinic setting given. In education. In foster care. | 1F | 1Female (F)  0Male (M) |
| (Gaynor & Harris, 2008) | Case studies | Depression | Depressed according to Diagnostic Interview for Children and BDI-II score (cuts off not given). Exclusion criteria OCD, psychotic, ASDs, acute suicidality. | Recruited from community using advertisements. 6/10 met inclusion criteria, 2/6 dropped out after 1st session, 4/6 completed intervention. | USA. 2 European American, 1 African American, 1 mixed ethnicity (European-Korean) | 4 (2F) | 2F 1M |
| (Weersing, Gonzalez, Campo, & Lucas, 2008) | Case studies | Anxiety or depression | Aged 7-17; met criteria for depression (major, minor or dysthymia) or anxiety (separation, generalized anxiety, social phobia, specific phobia); and no bipolar/psychosis, suicidal ideation, PTSD, intellectual disability, substance use, physical ill health or living without legal guardian. | Pediatric clinics | USA | 2 (1F) | 2F 0M |
| (Chu, Colognori, Weissman, & Bannon, 2009) | Case series | Anxiety (GAD, social phobia) and depression | Met DSM-IV for depression or anxiety based on Anxiety Disorders Interview Schedule for DSM-IV - Child Interview. No severe suicidal intent or behavior. | School counsellors at large state middle school identified and suggested students. 9/12 met criteria, 8 invited to participate, 5/6 who agreed to take part enrolled (1 moved away). | USA - 2 White, 1 African-American, one African immigrant, one Asian-American. | 5 (4 completed) (3F) | 3F 2M |
| (McCauley, Schloredt, Gudmundsen, Martell, & Dimidjian, 2011) | Illustrative case description, case descriptive composite but scores from one individual. | Depression - Major depressive disorder | None. Client diagnosed with major depressive disorder. | Selected clinical case - referred by primary care. | USA. | 1M | 0F 1M |
| (Wallis, Roeger, Milan, Walmsley, & Allison, 2012) | Case series | Depression | Referred with depression, scoring in depression range on CES-D | 10 recruited following referrals to local mental health service. 5 completed the study. | Australia. Adolescents in rural area. | 5F | 5F 0M |
| (Jacob, Keeley, Ritschel, & Craighead, 2013) | Case series | Depression - Major depressive disorder | MDD rated on the K-SADS and African American. 13-17 years. CDRS at least 45. Exclude if major mental health or developmental disorder, unwilling to attend, medication (if any) is stable for 2 months. | Recruited from urban hospital treating primarily low-income, African America patients. 10 screened, 4 not meet criteria, 3 withdrew (starting medication, suspected psychosis, not able make contract). | USA. Low-income Africa American adolescents. | 3 (1F) | 1F 2M |
| (Riley & Gaynor, 2014) | Case series | Depression | Met criteria for depression, no severe psychopathology or psychoactive medication use. | School district | USA | 11 (2F). 7 received BA. | 2F 5M |
| (Chu, Hoffman, Johns, Reyes-Portillo, & Hansford, 2015) | Case series (pilot study, reported as individual cases) | Anxiety (social anxiety; social anxiety and generalized anxiety disorder; social anxiety, separation anxiety and generalized anxiety disorder) and depression, relating to bullying | Bullying related distress and continuing to show clinical levels of low mood and anxiety | Referred to school counselling | USA | 5(1F) | 1F 4M |
| (Pass, Brisco, & Reynolds, 2015) | Case study | Depression | None. Depression presentation. | Selected clinical case - referred via CAMHS. | UK. | 1F | 1F 0M |
| (Pass, Whitney, & Reynolds, 2016) | Case study | Depression, in context risk | None. Adolescent with depression, with self-harm and fatigue. | Case attending clinic. | UK. | 1F | 1F 0M |
| (Pass, Hodgson, Whitney, & Reynolds, 2017) | Case study | Depression. | None. Adolescent with depression, self-harm and suicidal ideation. | Case attending clinic | UK. | 1F | 1F 0M |
| (Ritschel, Ramirez, Jones, & Craighead, 2011) | Pilot, A-B pre/post | Depression - Major depressive disorder | Diagnosis Major Depressive Disorder, based on K-SADS and CDRS-R scores (greater 65th percentile). Excluded if on psychotropic medication, other serious psychiatric diagnosis or autism, substance or alcohol use, estimated full-scale IQ lower than 80. | Recruited via advertisement in public places and radio advert. 7/14 screened were eligible. 1 dropped out - no longer interested. 5 completed BA. | USA. | 6 (3F) | 3F 3M |
| (Ritschel, Ramirez, Cooley, & Craighead, 2016) | Pre-post-follow-up | Depression - Major depressive disorder | Meet criteria for MDD using K-SADS and greater than 65th percentile scores on CDRS-R. No current psychotropic medication (except ADHD medications), no current/past diagnosis of bipolar, psychotic type disorder, pervasive development disorder, no current conduct disorder, life-threatening anorexia, OCD, autism, substance misuse in last 3 months and IQ must be above 80. | Radio advertising, posters around schools and public areas. Also screened callers to their psychology clinic. 28/65 eligible. | USA. Ethnically diverse sample. | 28 (22/28 completed the study, 17/22 3 month follow-up, 16/22 6 month follow-up). (19F) | 19F 9M |
| (Pass, Lejuez, & Reynolds, 2017) | Pre-post | Depression. | No formal criteria – referred for treatment at outpatient clinic and experiencing depression symptoms. | Cases referred to outpatient clinic. | UK. | 20 (18/20 completed treatment). (18F) | 18F 2M |
| (Chu et al., 2016) | RCT | Anxiety or depression or both | Current clinical or subclinical diagnosis of depression (any type) or anxiety (generalized anxiety disorder, social phobia, separation anxiety). Excluded if neither depression nor anxiety as principle diagnosis, pervasive developmental disorder, serious mental illness such as schizophrenia, high risk/past year suicidality. If learning disability, excluded if poor fit to the group context. ADIS (for anxiety) and CGI-S (for depression) used. | Recruited from school. 895 were screened. 50 completed diagnostic interview. 35 included and randomized. | USA. Ethnically diverse sample (13 Hispanic, 15 African-American, 5 White non-Hispanic, 2 multiple) with broad range of family incomes. | 35 (16/21 completed BA intervention, 12/14 completed wait-list) (25F) | 25F 10M |
| (McCauley et al., 2016) | RCT | Depression | Age 12 to 18, one parent/guardian willing to participate, depression meeting DSM-IV criteria (using CDRS-R and SMFQ), willingness to be randomized. No psychosis or mania, substance use, suicidality or acute medical illness. | Recruited from primary care and mental health care providers in metropolitan area. 124 screened, 60 eligible. | USA. Metropolitan area. Recruited via primary care and mental health. | 60 (124 screened, 85 completed baseline, 60 randomized, 29/35 received BA, 24/25 received other evidence based intervention, 27/35 completed BA 12 month follow-up, 16/25 completed control arm 12 month follow-up.) (38F) | 22F 13M |
| (Takagaki, Okamoto, et al., 2016) and (Takagaki, Jinnin, et al., 2016) and (Mori et al., 2016) | RCT | Depression - sub clinical threshold. | BDI-II score greater than or equal to 10, then completion of CIDI leading to diagnosis of sub-clinical threshold for depression. No acute suicide risk. | Recruited from first year university students. 208/428 contacted consented to participate | Japan. University (first year students). | 62 in BA arm, 56 in control arm (118 were randomized. BA retention to post-assessment was 61/62 and control group was 55/56) (45F) | 24F 38M |
| (Weersing et al., 2017) | RCT | Anxiety or depression | As for (Weersing et al., 2008) | Recruited through pediatric clinics. 518 screened, 397 eligible, 290 completed baseline, (101 not meet criteria, 4 declined), 185 randomized. | USA. | 95 in BA, 90 in control. BA retention to end of treatment 86/95, control group retention to end of treatment 79/90). 107F | 54F  41M |

**Online Resource 2 Outcomes, Time points, Comparator, Analysis and Results of studies**

| **Study reference** | **Primary outcome measures** | **Data collection points** | **Comparator** | **Analysis** | **Summary result** |
| --- | --- | --- | --- | --- | --- |
| (Ruggiero et al., 2007) | BDI | Pre, session 6/8 BATD, last session. | None | Pre-post single case - descriptive only | BDI fell from 13 (mild) at baseline to 2 (non-clinical) at end of final session. |
| (Gaynor & Harris, 2008) | DISC and BDI-II | Baseline, immediately post-treatment and follow-up (varied by participant from 1 to 3 months). BDI-II also at every session. | None | Pre-post and comparisons to measure cut-off values. | All 4 participants no longer met DISC criteria for MDD and BDI-II's fell to non-depressed range by post-treatment. Inconsistent results for mediator role of behavior change or beliefs. |
| (Weersing et al., 2008) | SCARED, CDI | Pre treatment, end treatment (8 weeks), 12 week post treatment, 24 week follow-up. | None | Case data reported in graph and narrated without statistics. | End of treatment (week 8) - clinically significant reduction in anxiety not depression. Week 12 follow-up - clinically significant reduction maintained for anxiety and achieved for depression, both retained for 24 week follow-up. |
| (Chu et al., 2009) | ADIS-IV-C, MASC-C/P, CESD-C/P | Pre and post. | None | Pre-post comparisons as group means. Case studies report change on diagnostic criteria. | ADIS CSR pre-treatment mean 6.00 (s.d. 0.71) to 3.25 (s.d. 1.26). CGI-S from 5.60 (s.d. 0.55) to 4.00 (s.d. 1.41). MASC and CESD symptom reporting mean drops 12.58 and 8.83 points respectively from pre to post-treatment. Post-test, 3/4 no longer met diagnostic criteria for principle diagnosis nor secondary diagnosis. |
| (McCauley et al., 2011) | CDRS-R and SMFQ | Pre and then repeated, but score details for after baseline not given. | None | None - brief description only. Post-test scores not given | CDRS-R 79 at baseline. SMFQ 18 at baseline. Scores never fell into non-depressed range |
| (Wallis et al., 2012) | BDI | Baseline, 2 weeks, 3 weeks, 6 weeks and end of treatment (10 weeks) | None | Graphed only. | All reduced levels of depression by follow-up. Means are not given but are estimated from graph: baseline mean 26 (s.d. 10) to end of intervention mean 13 (s.d. 7). At baseline, all in mild-moderate range of depression. By follow-up 2 below clinical range, 3 in mild range. |
| (Jacob et al., 2013) | K-SADS, CDRS-R, BDI-II, CGI-S | Baseline, end of treatment. CDRS-R at 9 weeks also and weekly BDI-II. | None | Means for 3 cases reported pre-post. Individual scores reported also. | CDRS-R mean fell from 59.3 (13.6) to 33.0 (19.1). BDI-II from 21.7 (4.1) to 4.0 (2.0). CGI-S from 4.7 (1.2) to 2.0 (1.7). K-SADS indicated all three had MDD at pre-test, but only 1 met MDD criteria at post-test. |
| (Riley & Gaynor, 2014) | CDRS-R CDI | Pre-, post-control treatment, post BA, 2 month follow-up | First completed 3 sessions of non-directive therapy. If no clinically significant change, then offered BA. | Participants receiving BA had all received non-directive therapy first - comparisons of end of control to end of BA made. | Pre-BA to post-BA revealed significant difference in CDRS-R and CDI scores (Z=-2.37, p<0.02). 4/7 clinically significant reduction in depression symptoms at post-BT. Data from 2 month follow-up not presented for group. Inconsistent findings regarding relevance of technique use or behavior change as a mediator of change in depression. |
| (Chu et al., 2015) | ADIS-IV-C to create CSR. SCARED, CES-D | Baseline and post-treatment (interval not given) | None | Pre-post compared for each individual. | CSR reduced from clinical to non-clinical for 4 of 6 participants on at least one diagnostic category. SCARED scores significantly reduced for 2/5 participants, with CES-D significantly reduced for 3/5. |
| (Pass et al., 2015) | RCADS, RCADS-P (parent version) | Session by session and 1 month follow-up. | None | Not provided, other than in graphical form. | Results reported as graphs. “Reliable improvement” in RCADS scales seen. |
| (Pass et al., 2016) | RCADS, RCADS-P (parent version) | Session by session and 6 week review. | None | Session by session scores graphed only. | Results reported as graphs. Change clinically significant. RCADS depression changed from 19 at assessment to 11 at review (cut-off for normal range is 12 or below). RCADS-P depression score decreased from 18 at assessment to 6 at review (cut-off for normal range is 8 or below). |
| (Pass, Hodgson, et al., 2017) | RCADS, RCADS-P (parent version) | Session by session and 1 month follow-up. | None | Session by session scores graphed only. | Results reported as graphs. Change clinically significant. RCADS depression self-report changed from above clinical cut off to below by session 4 and remained below clinical cut-off from then. Parent initially in clinical range and fell report below clinical range at review session. |
| (Ritschel et al., 2011) | K-SADS, CDRS-R, BDI-II | First session and posttreatment (interval not given, appears to be last treatment session). BDI also weekly. | None | Pre-post | CDRS pre mean 57.67 (s.d. 11.18), post 27.67 (80.7), significant at p<0.01. BDI pre-intervention 28.00 (6.51), fell to 6.00 (5.87) at post, significant at p<0.001. 4 of 6 participants moved from "depressed" range to "normal" range at post-test. |
| (Ritschel et al., 2016) | CDRS-R, BDI-II, CBCL and CGI-S | Baseline, mid-point, end of treatment, 3-month follow-up, 6 month follow-up | None | Pre-post analyses completed with relevant ANOVA. Impact of activation on outcomes examined using regression. | End of treatment, 90.9% of completers no longer met criteria for MDD - 54.5% fully remitted, 36.4% responders, 9.1% non-responders. Significant differences between baseline and end of treatment in CDRS-R, BDI-II, CGI-S, CBCL. For CDRS-R and BDI-II effect size (partial eta square) was 0.63. Statistically significant decreases in CDRS-R, BDI-II and CGI-S occurred between baseline and midpoint. Concurrently with reduced depression symptoms were increases in CHS and BADS. Maintenance of effect was examined by repeated measures ANOVA for 6-month follow-up. No significant effect of time - indicating that changes were maintained. BADS scored were predictive of lower end of treatment depression, when controlling for baseline depression. |
| (Pass, Lejuez, et al., 2017) | RCADS, RCADS-P (parent version) | Pre, post (end of session 8) and 1 month follow-up. (Session by session also recorded for RCADS depression) | None | Assessment -session 8 and Assessment – 1 month review paired analyses completed. Intention to treat approach used. | RCADS Depression scores pre mean 20.65 (s.d. 5.66), post mean 15.00 (s.d. 6.79), 1 month follow-up 14.45 (s.d. 7.98). Pre-post comparison p<0.01, d=0.90; Pre-follow-up comparison p<0.01, d=0.90. Shift from clinical to non-clinical range.  RCADS depression parents pre 16.58 (5.85), post 11.63 (3.95), follow-up 10.66 (6.52). Pre-post p<0.01, d=0.99; pre-follow-up p<0.01, d=0.96 . No shift from clinical range.  Clinically significant change on the RCADS was observed for 6 participants between pre and post time points and for 7 between pre and follow-up. |
| (Chu et al., 2016) | ADIS-IV, CDRS-R, CGI-S, SCARED, CES-D. | Pre, post, 4 month follow-up. | Wait-list control with no contact | Intention to treat and mixed effects modelling comparing pre-post and pre-post-4 month follow-up. | Improvement in CGI-S compared to waiting list - more remission primary diagnosis (OR3.33) and for secondary diagnosis (OR 21.6). ADIS revealed significantly lower severity in secondary diagnosis only (beta = -2.09). Reduction in co-morbid diagnoses. Symptom scores (CDRS-R, SCARED and CES-D) were not significantly different at post-treatment between waiting list and treatment group. Significant trends (p<0.05) indicating maintained score improvement for pre-post-4 month scores on CGI-S, ADIS-IV, and SCARED but not CESD. BADS-A not significantly associated treatment by time point effect at p<0.05. |
| (McCauley et al., 2016) | CDRS-R, CGI-S, SMFQ | Pre-, end of treatment, 6 month follow-up, 12 month follow-up | Evidence based intervention for up to 14 sessions - 21 received CBT, 4 received interpersonal psychotherapy. | Comparisons between the groups using ANOVA analyses. Intention to treat analysis. | Both groups showed significant decrease in symptoms, but not different between the groups. No significant change in activation over time, but rumination decreased in both BA and control conditions. |
| (Takagaki, Okamoto, et al., 2016) and (Takagaki, Jinnin, et al., 2016) and (Mori et al., 2016) | BDI-II, Japanese version | Pre-post | Control received no treatment nor contact within the 5 week intervention period. | Comparisons between the groups using ANOVA analyses. Intention to treat analysis. | Treatment group improved significantly more than control from baseline to post-intervention on BDI-II - effect size between groups was 0.90 (from ANOVA). There was a significant intervention between the groups on the BADS also: higher activation in the BA group. Comparing the 21 who had only individual and the 20 who had only group, there was no significant differences in outcomes.  Activation did not have a direct effect on depression symptoms (estimated direct effect -0.21, 95%CI -0.52 to 0.07). Activation changes had effect on change in reinforcement (-0.15, 95%CI 0.11 to 0.54). Change in reinforcement had a direct effect on change in depression (-0.43, 95%CI -0.86 to -0.01). Reinforcement change mediated the effect on change in activation on change in depression - indirect effect estimated to be -0.15, 95%CI -0.33 to -0.03. |
| (Weersing et al., 2017) | CGI | Baseline, post intervention at week 16. | Referrals to relevant service with problem solving re: barriers to treatment. | ANOVA with Number Needed to Treat (NNT) | 50/88 receiving some BA no longer clinically significant CGI Improvement scores at week 16, compared 20/71 controls (chi squared 13.09, p<0.01). NNT 4, 95%CI 2.3-7.2. CGI Severity scores for control group from 4.1 (0.8) at baseline to 3.4 (1.3) at week 16, with 4.2 (0.80) to 2.6 (1.2) for BA group. Effect size (d) comparing two groups at week 16 = 0.642. |

**BDI** - Beck Depression Inventory (Beck, Ward, Mendelson, Mock, & Erbaugh, 1961); **BDI-II** - Beck Depression Inventory – II (Beck, Steer, & Brown, 1996), with the Japanese version (Kojima & Furukawa, 2003); **DISC** - Diagnostic Interview Schedule for Children (Shaffer, Fisher, Lucas, Dulcan, & Schwab-Stone, 2000); **ADIS-IV-C** - Anxiety Disorders Interview Schedule for DSM-IV – Child Interview, used to create **CSR** - Clinical Severity Rating (Silverman & Albano, 1996); **MASC-C/P** - Multidimensional anxiety scale for children – Child and parent scale (March, Parker, Sullivan, Stallings, & Conners, 1997); **CESD-C/P** - Centre for Epidemiologic Studies Depression Scale for Children – Child / Parent reports (Radloff, 1977); **CDRS-R** - Children’s Depression Rating Scale – Revised (Poznanski & Mokros, 1996); **SMFQ** - Short Moods and Feelings Questionnaire (Angold, Costello, Messer, & Pickles, 1995); **KSADS** - Kiddie Schedule for Affective Disorders and Schizophrenia (Kaufman et al., 1997); **CGI-I/S** - Clinical Global Impressions Scale – Impairment / Severity (Rapoport & Conners, 1985); **SCARED** - Screen for childhood anxiety related emotional disorders (Birmaher et al., 1997); **RCADS** - Revised Child Anxiety and Depression Scale, including RCADS-P as parent version (Chorpita, Yim, Moffitt, Umemoto, & Francis, 2000); and **CBCL** Child Behavior Checklist for Ages 6-18 (Achenbach & Rescorla, 2001).

**Online Resource 3 Effect sizes for RCT studies**

| **Study reference** | **Measure** | **Effect size (Hedges g)** | **95% confidence interval** |
| --- | --- | --- | --- |
| (Chu et al., 2016) | CDRS-R | 0.99 | -5.35 to 7.32 |
|  | CGI-S | 1.205 | 0.87 to 1.59* |
|  | CSR Principal diagnosis | 0.63 | -0.04 to 1.30 |
|  | CESD – Parent | 0.06 | -2.02 to 2.15 |
|  | CESD – Youth | 0.24 | -3.42 to 3.90 |
|  | SCARED – Parent | 0.54 | -2.68 to 3.76 |
|  | SCARED – Youth | 0.42 | -4.13 to 4.97 |
|  | BADS-A – Parent (combining avoidance and activation) | -0.35 | -4.30 to 3.60 |
|  | BADS-A – Youth (combining avoidance and activation) | -0.52 | -6.16 to 5.11 |
| (McCauley et al., 2016) | CDRS-R | -0.35 | -3.89 to 3.20 |
|  | CGI-S | -0.08 | -0.40 to 0.24 |
|  | SMFQ | -0.03 | -1.81 to 1.75 |
|  | BAD-S Activation | 0.09 | -2.69 to 2.87 |
|  | BAD-S Avoidance | -0.07 | -2.77 to 2.62 |
| (Takagaki, Okamoto, et al., 2016) | BDI | -0.90 | -1.28 to 0.52 |
|  | BADS Total | 0.375 | -2.40 to 3.15 |
|  | BADS Activation | 0.65 | -0.57 to 1.87 |
|  | BADS Avoidance | -0.37 | -1.53 to 0.80 |
| (Weersing et al., 2017) | CDRS-R | 0.31 | -1.02 to 1.64 |
|  | CGI-S | 0.639 | 0.446 to 0.833* |
|  | CGI-I | 0.67 | 0.48 to 0.85* |
|  | CGAS | -0.58 | -2.34 to 1.17 |

*95% confidence interval of effect size does not include zero: statistical significance at p<0.05 assumed.

**Online Resource 4 Summary content of BA interventions**

|  | **Study** | **Activity monitoring** | **Activity scheduling** | **Contingency management** | **Values & goals assessment** | **Skills training** | **Relaxation** | **Targeting verbal behaviors** | **Avoidance** | **Psycho-education** | **Functional analysis** | **Relapse prevention** | **Other** |
| --- | --- | --- | --- | --- | --- | --- | --- | --- | --- | --- | --- | --- | --- |
|  | (Ruggiero et al., 2007) | X | X |  | X |  |  |  |  |  | X |  |  |
|  | (Gaynor & Harris, 2008) | X | X |  | X | X PS |  |  |  | X BA, depression | X |  |  |
| * | (Weersing et al., 2008) | X | X |  |  | X PS | X |  | X | X anxiety, depression |  | X |  |
| # | (Chu et al., 2009) | X | X |  | X | X PS |  |  | X TRAP/TRAC |  | X |  | Motivational interviewing (MI)  Graded exposure |
| + | (McCauley et al., 2011) | X | X |  | X | X PS |  | X | X |  | X |  | MI |
|  | (Wallis et al., 2012) | X | X |  | X |  |  |  |  | X BA |  |  |  |
| ⁑ | (Jacob et al., 2013) | X | X |  | X | X PS |  |  | X TRAP/TRAC | X BA, depression | X | X |  |
|  | (Riley & Gaynor, 2014) |  | X | X |  | X PS |  |  |  |  |  |  |  |
| # | (Chu et al., 2015) | X | X |  | X | X PS, social skills |  |  | X TRAP/TRAC | X mood, bullying | X |  | MI |
| ⁂ | (Pass et al., 2015) | X | X |  | X | X PS |  |  |  | X BA, depression | X | X |  |
| ⁂ | (Pass et al., 2016) | X | X |  | X | X PS |  |  |  | X BA | X | X |  |
| ⁂ | (Pass, Hodgson, et al., 2017) | X | X |  | X | X PS |  |  |  | X BA | X | X |  |
| ⁑ | (Ritschel et al., 2011) | X | X |  | X | X PS |  | X |  | X BA, depression | X | X |  |
| ⁑ | (Ritschel et al., 2016) | X | X |  | X | X PS |  | X |  | X BA, depression | X | X |  |
| ⁂ | (Pass, Lejuez, et al., 2017) | X | X |  | X | X PS |  |  |  | X BA, depression | X | X |  |
| # | (Chu et al., 2016) | X | X |  | X | X PS |  |  | X TRAP/TRAC |  | X | X | MI  Graded exposure |
| + | (McCauley et al., 2016) | X | X |  | X | X PS |  | X | X | X BA | X | X | MI |
|  | (Mori et al., 2016; Takagaki, Jinnin, et al., 2016; Takagaki, Okamoto, et al., 2016) |  | X |  | X | X PS |  | X | X | X BA, depression |  |  |  |
| * | (Weersing et al., 2017) | X | X |  |  | X PS | X |  | X | X anxiety, depression |  | X |  |

Key – Matching symbols to left of reference indicate where the studies use the same intervention content.

**Online Resource 5 Details of Intervention Delivery**

| **Study** | **Location of delivery** | **Therapist** | **Mode of delivery*** | **Number, timing and attendees of sessions** |
| --- | --- | --- | --- | --- |
| (Ruggiero et al., 2007) | Outpatient | Male, graduate student trained in CBT | One-to-one | 10 in total. 2 communication skills session, 1 joint with foster mother. 8 BATD sessions with young person. Weekly. |
| (Gaynor & Harris, 2008) | Outpatient clinic | Female Clinical Psychology doctorate student | One-to-one | 12 individual +1 or 2 with parent. Sessions 1 hour. Twice weekly for first 4 sessions, then weekly. This number does not include what the authors refer to as "baseline" or "assessment" sessions. |
| (Weersing et al., 2008) | Primary care pediatric clinic | On-site Mental health clinical staff, previously attended CBT workshops. 2 day training by Clinical Psychologist in BA. 1 supervised training case. | One-to-one | 8 45 minute sessions over 12 weeks |
| (Chu et al., 2009) | School | Trained mental health specialist - Clinical Psychologist with 13 years’ experience and co-led by 4th year female clinical psychology doctoral student with extensive CBT training. | Group | 13 weekly group sessions, length of school lesson - approximately 40 minutes. |
| (McCauley et al., 2011) | Outpatient clinic | Female, trained "therapist" | One-to-one | 14 individual sessions over 12 weeks, typically with young person alone. |
| (Wallis et al., 2012) | Outpatient | Social workers with brief training in BA | One-to-one | 10 sessions with individual, 1 per week |
| (Jacob et al., 2013) | Outpatient clinic | Clinical staff trained in BA by Dimidijian. Intensive and ongoing supervision - weekly. | One-to-one | Invited to 14 - 17 over 6 months. Attended 17, 15 and 14. Typically with individual but with parents also at request. |
| (Riley & Gaynor, 2014) | Predominately at school, where not possible then university rooms used. | Doctoral level student in Clinical Psychology | One-to-one | 9 in total. 3 sessions each for child, carer, and both together. |
| (Chu et al., 2015) | School | Graduate Psychology students, experienced in CBT | Group | 14 38 minute sessions with young people - to fit within school lesson time. Weekly |
| (Pass et al., 2015) | Outpatient CAMHS. | Female clinical psychologist. | One-to-one | 9 session: 8 sessions (60 mins), approximately weekly, with one review (30 mins, 1 month later). Parents invited to 3 sessions. |
| (Pass et al., 2016) | Outpatient clinic | Clinical Psychologist. Trained in BATD by Lejeuz | One-to-one | 9 sessions. Mostly individual sessions, some with mother. Duration not explicitly stated |
| (Pass, Hodgson, et al., 2017) | Outpatient clinic | Psychology assistant without specialist clinical training.  Trained in BATD by Lejeuz | One-to-one | 8 weekly one hour sessions, mother also attended 3. |
| (Ritschel et al., 2011) | Outpatient clinic | 2 doctoral level faculty members and one senior-level graduate student from the mood disorders research group. | One-to-one | 22 maximum over 18 weeks. Attendance mean 14 session (s.d.2) range 11 - 16 sessions. Typically individual sessions. |
| (Ritschel et al., 2016) | Outpatient clinic | Three doctoral level psychologists, two advanced graduate students. Additional experienced therapist as consultant. 2 days of training from Dimidjian. | One-to-one | 22 maximum over 18 weeks. Typically individual sessions - young person could invite others. Session duration not given. |
| (Pass, Lejuez, et al., 2017) | Outpatient clinic | Psychology assistants and Clinical Psychologist. Trained in BATD by Lejeuz. | One-to-one | 6-8 one hour weekly one hour sessions. Parents invited to 3 sessions. One further review session. |
| (Chu et al., 2016) | School | Two therapists lead each group. Study used total of seven therapists. One clinical psychologist, four psychology graduate students, two school counsellors. 5 female. 2 Caucasian, two Hispanic, one African-American, one Asian American. | Group and 2 one-to-one sessions | Planned 10 weekly (delivered 12-15), hour long sessions with group (7 young people) plus two individual sessions. |
| (McCauley et al., 2016) | Outpatient clinic | Post-doctoral fellows, psychology faculty and a social worker. | One-to-one | 14 sessions over 12 weeks, typically with individual. (No further details given). |
| (Mori et al., 2016; Takagaki, Jinnin, et al., 2016; Takagaki, Okamoto, et al., 2016) | Outpatient clinic | Male doctoral research fellow with CBT experience. Trained with practice cases (completed whole intervention) and ongoing supervision, with recorded sessions. | Small groups - between 1 and 3 per session. (49.4% sessions were one-to-one) | 5 weekly, one hour sessions |
| (Weersing et al., 2017) | Primary care pediatric clinic | Master’s level therapists trained 0.5days in manual and 2 supervised training cases. | One-to-one | 8-12 45-minute sessions over 16 weeks |

*All interventions were delivered face to face

**References used in supplementary information**

(all present in main article, presented here for ease of reference)

Achenbach, T., & Rescorla, L. (2001). Manual for the Achenbach system of empirically based assessment school-age forms profiles. In. Burlington, VT: Aseba.

Angold, A., Costello, E. J., Messer, S. C., & Pickles, A. (1995). Development of a short questionnaire for use in epidemiological studies of depression in children and adolescents. *International journal of methods in psychiatric research*.

Beck, A. T., Steer, R. A., & Brown, G. K. (1996). Beck depression inventory-II. *San Antonio, 78*(2), 490-498.

Beck, A. T., Ward, C. H., Mendelson, M., Mock, J., & Erbaugh, J. (1961). An inventory for measuring depression. *Archives of General Psychiatry, 4*(6), 561-571.

Birmaher, B., Khetarpal, S., Brent, D., Cully, M., Balach, L., Kaufman, J., & Neer, S. M. (1997). The screen for child anxiety related emotional disorders (SCARED): scale construction and psychometric characteristics. *Journal of the American Academy of Child & Adolescent Psychiatry, 36*(4), 545-553.

Chorpita, B. F., Yim, L., Moffitt, C., Umemoto, L. A., & Francis, S. E. (2000). Assessment of symptoms of DSM-IV anxiety and depression in children: A revised child anxiety and depression scale. *Behaviour Research and Therapy, 38*(8), 835-855.

Chu, B. C., Colognori, D., Weissman, A. S., & Bannon, K. (2009). An Initial Description and Pilot of Group Behavioral Activation Therapy for Anxious and Depressed Youth. *Cognitive and Behavioral Practice, 16*(4), 408-419.

Chu, B. C., Crocco, S. T., Esseling, P., Areizaga, M. J., Lindner, A. M., & Skriner, L. C. (2016). Transdiagnostic group behavioral activation and exposure therapy for youth anxiety and depression: Initial randomized controlled trial. *Behaviour Research and Therapy, 76*, 65-75.

Chu, B. C., Hoffman, L., Johns, A., Reyes-Portillo, J., & Hansford, A. (2015). Transdiagnostic Behavior Therapy for Bullying-Related Anxiety and Depression: Initial Development and Pilot Study. *Cognitive and Behavioral Practice, 22*(4), 415-429.

Gaynor, S. T., & Harris, A. (2008). Single-participant assessment of treatment mediators: Strategy description and examples from a behavioral activation intervention for depressed adolescents. *Behavior Modification, 32*(3), 372-402.

Jacob, M., Keeley, M. L., Ritschel, L., & Craighead, W. E. (2013). Behavioural activation for the treatment of low-income, African American adolescents with major depressive disorder: a case series. *Clinical Psychology & Psychotherapy, 20*(1), 87-96.

Kaufman, J., Birmaher, B., Brent, D., Rao, U., Flynn, C., Moreci, P., . . . Ryan, N. (1997). Schedule for affective disorders and schizophrenia for school-age children-present and lifetime version (K-SADS-PL): initial reliability and validity data. *Journal of the American Academy of Child & Adolescent Psychiatry, 36*(7), 980-988.

Kojima, M., & Furukawa, T. A. (2003). Japanese Version of the Beck Depression Inventory. In. Tokyo: Nippon-Hyoron-sha Co.

March, J. S., Parker, J. D., Sullivan, K., Stallings, P., & Conners, C. K. (1997). The Multidimensional Anxiety Scale for Children (MASC): factor structure, reliability, and validity. *Journal of the American Academy of Child & Adolescent Psychiatry, 36*(4), 554-565.

McCauley, E., Gudmundsen, G., Schloredt, K., Martell, C., Rhew, I., Hubley, S., & Dimidjian, S. (2016). The Adolescent Behavioral Activation Program: Adapting Behavioral Activation as a Treatment for Depression in Adolescence. *Journal of Clinical Child & Adolescent Psychology, 45*(3), 291-304.

McCauley, E., Schloredt, K., Gudmundsen, G., Martell, C., & Dimidjian, S. (2011). Expanding behavioral activation to depressed adolescents: Lessons learned in treatment development. *Cognitive and Behavioral Practice, 18*(3), 371-383.

Mori, A., Okamoto, Y., Okada, G., Takagaki, K., Jinnin, R., Takamura, M., . . . Yamawaki, S. (2016). Behavioral activation can normalize neural hypoactivation in subthreshold depression during a monetary incentive delay task. *Journal of Affective Disorders, 189*, 254-262.

Pass, L., Brisco, G., & Reynolds, S. (2015). Adapting brief Behavioural Activation (BA) for adolescent depression: A case example. *the Cognitive Behaviour Therapist, 8*.

Pass, L., Hodgson, E., Whitney, H., & Reynolds, S. (2017). Brief behavioral activation treatment for depressed adolescents delivered by nonspecialist clinicians: A case illustration. *Cognitive and Behavioral Practice*, No Pagination Specified. doi:10.1016/j.cbpra.2017.05.003

Pass, L., Lejuez, C. W., & Reynolds, S. (2017). Brief behavioural activation (Brief BA) for adolescent depression: A pilot study. *Behavioural and Cognitive Psychotherapy*, No Pagination Specified-No Pagination Specified. doi:10.1017/S1352465817000443

Pass, L., Whitney, H., & Reynolds, S. (2016). Brief Behavioral Activation for Adolescent Depression: Working with complexity and risk. *Clinical Case Studies, 15*(5), 360-375.

Poznanski, E. O., & Mokros, H. B. (1996). *Children's depression rating scale, revised (CDRS-R)*. Los Angeles: Western Psychological Services

Radloff, L. S. (1977). The CES-D scale: A self-report depression scale for research in the general population. *Applied psychological measurement, 1*(3), 385-401.

Rapoport, J., & Conners, C. (1985). Rating scales and assessment instruments for use in pediatric psychopharmacology research - introduction In. Washington, US: National Institute of Mental Health.

Riley, A. R., & Gaynor, S. T. (2014). Identifying mechanisms of change: Utilizing single-participant methodology to better understand behavior therapy for child depression [Press release]

Ritschel, L. A., Ramirez, C. L., Cooley, J. L., & Craighead, W. (2016). Behavioral activation for major depression in adolescents: Results from a pilot study. *Clinical Psychology: Science and Practice, 23*(1), 39-57.

Ritschel, L. A., Ramirez, C. L., Jones, M., & Craighead, W. (2011). Behavioral activation for depressed teens: A pilot study. *Cognitive and Behavioral Practice, 18*(2), 281-299.

Ruggiero, K. J., Morris, T. L., Hopko, D. R., & Lejuez, C. W. (2007). Application of behavioral activation treatment for depression to an adolescent with a history of child maltreatment. *Clinical Case Studies, 6*(1), 64-78.

Shaffer, D., Fisher, P., Lucas, C. P., Dulcan, M. K., & Schwab-Stone, M. E. (2000). NIMH Diagnostic Interview Schedule for Children Version IV (NIMH DISC-IV): description, differences from previous versions, and reliability of some common diagnoses. *Journal of the American Academy of Child & Adolescent Psychiatry, 39*(1), 28-38.

Silverman, W. K., & Albano, A. M. (1996). *Anxiety Disorders Interview Schedule for DSM-IV.: Child interview schedule* (Vol. 2): Graywind Publications.

Takagaki, K., Jinnin, R., Mori, A., Nishiyama, Y., Yamamura, T., Yokoyama, S., . . . Yamawaki, S. (2016). Mechanisms of behavioral activation for late adolescents: Positive reinforcement mediate the relationship between activation and depressive symptoms from pre-treatment to post-treatment. *Journal of Affective Disorders, 204*, 70-73.

Takagaki, K., Okamoto, Y., Jinnin, R., Mori, A., Nishiyama, Y., Yamamura, T., . . . Yamawaki, S. (2016). Behavioral activation for late adolescents with subthreshold depression: A randomized controlled trial. *European Child & Adolescent Psychiatry Mar*(Pagination), No Pagination Specified.

Wallis, A., Roeger, L., Milan, S., Walmsley, C., & Allison, S. (2012). Behavioural activation for the treatment of rural adolescents with depression. *The Australian Journal Of Rural Health, 20*(2), 95-96.

Weersing, V. R., Brent, D. A., Rozenman, M. S., Gonzalez, A., Jeffreys, M., Dickerson, J. F., . . . Iyengar, S. (2017). Brief behavioral therapy for pediatric anxiety and depression in primary care: A randomized clinical trial. *JAMA psychiatry, 74*(6), 571-578. doi:10.1001/jamapsychiatry.2017.0429

Weersing, V. R., Gonzalez, A., Campo, J. V., & Lucas, A. N. (2008). Brief behavioral therapy for pediatric anxiety and depression: Piloting an integrated treatment approach [Press release]
